# Lydston’s Rejected Letter

**Published:** 1905-08

**Authors:** 


### Lydston's Rejected Letter.

### Is the Journal of the American Medical Association a Partisan Organ?

The letter published herewith was sent to the Journal of the A. M. A. for publication, and was returned, with the explanation that the editor did "not see fit to publish it" without alteration. He failed to inform me whether he took exception to the style or to the subject matter. I saved my ego center from shock by inferring that the subject matter was offensive. The distinguished editor of the *Journal* has adversely criticised my style on several occasions, and another blow would have given me literally a "finish." The criticisms of a master, while usually well meant, are often drastic medicine for budding aspirants for literary honors. Being curious to know how the rejected stuff would look in print, I have done the deed herewith in evidence. If it "takes," I am going to put a nice little hand press in my laboratory, where I can cultivate antitoxin for illiberalitis, for the inoculation of pachyderms, the macro-cephalus communis, acephalus vulgaris and other zoologic and pathologic things, and be certain that it will be seen, if not read. There will be no board of censors.

If the American Medical Association desires the sympathy and co-operation of the medical profession at large, it must avoid partisanry and affronts to the medical rank and file. I submit that the comments of the editor of the *Journal of the A. M. A.* mentioned in my letter, were an insult, not only to the general practitioner, but also to a large number of eminent men, whose contributions and pictures have appeared from time to time in the *Medical Brief*. Among these are some of the most distinguished men in America—men with whom anyone should be proud to associate.

G. FRANK LYDSTON.

---

DEAD SURE THING.—"To prevent hair falling off; only sure remedy known. Send $1. Surepop Co. Bible House, Chicago." Mrs. Sara Jane Gullible sent the dollar and received the following: "If your hair threatens to fall off *hold on with both hands.* Dollar received; thanks. Surepop Co. Next."

---

"WILL sell my eye and ear practice to registered physician or optician and guarantee $250 a month profit on refraction alone. $1000 cash necessary.

DR. J. T. S.,
302-517 S. Broadway, Los Angeles, Cal."

